# Standardized ileal digestible amino acids and net energy contents in full fat and defatted black soldier fly larvae meals (*Hermetia illucens*) fed to growing pigs

**DOI:** 10.1093/tas/txaa104

**Published:** 2020-06-29

**Authors:** Michelina Crosbie, Cuilan Zhu, Anna K Shoveller, Lee-Anne Huber

**Affiliations:** Department of Animal Biosciences, University of Guelph, Guelph, ON, Canada

**Keywords:** black soldier fly larvae meal, pig, net energy, standardized ileal digestible amino acids

## Abstract

Two experiments were conducted to determine standardized ileal digestibility (SID) of amino acids (AA; Exp. 1) and net energy (Exp. 2) in two black soldier fly larvae meal (BSFLM) samples [full fat (FF; 42.5% crude protein (CP), as-fed) and defatted (DF; 40.8% CP; as-fed)] for growing pigs. Two cornstarch-based diets were formulated with FF and DF BSFLM as the sole sources of AA. A nitrogen-free diet was also used, and the corn starch:sucrose:oil ratio was kept constant among diets to calculate digestible energy (DE) by difference method. In each experiment, pigs were fed 2.8 × estimated maintenance energy requirement. In Exp. 1, eight ileal-cannulated barrows (25.1 ± 0.41 kg initial body weight) were used in a replicated 2 × 2 Latin square design (*n* = 8). In each period, pigs were adapted to diets for 5 d followed by 2 d of continuous ileal digesta collection for 8 h. The SID of AA were calculated using basal endogenous losses for pigs fed a nitrogen-free diet. In Exp. 2, eight barrows [23.4 ± 0.54 kg initial body weight (BW)] were used in a partially replicated Latin square design (*n* = 8). In each period, pigs were adapted to diets for 7 d, followed by 5 d of total urine collection and fecal grab sampling. The SID of CP (80.6 ± 1.1%) and Lys (88.0 ± 1.4%) were not different between FF and DF BSFLM. The SID of Arg, Val, Ala, and Pro tended to be less, and the SID of Met tended to be greater for the FF versus the DF BSFLM (*P* = 0.034, 0.090, 0.053, 0.065, 0.074, respectively). Digestible energy (4,927 vs. 3,941 ± 75 kcal/kg), metabolizable energy (4,569 vs. 3,396 ± 102 kcal/kg), and predicted net energy (3,477 vs. 2,640 ± 30 kcal/kg, using equations from Noblet; 3,479 vs. 2,287 ± 28 kcal/kg, using equations from Blok, respectively) were greater for the FF versus the DF BSFLM (*P* < 0.05). The apparent total tract digestibility of neutral detergent fiber and acid detergent fiber were greater for the FF versus the DF BSFLM (*P* ≤ 0.05). Both FF and DF BSFLM had high SID for most AA; however, FF BSFLM was a better source of net energy for growing pigs. Therefore, both FF and DF BSFLM could be used as protein alternatives in growing pig diets.

## INTRODUCTION

Presently, many livestock diets rely on soybean meal (SBM) as a high-quality protein source, with 85% of the world’s soybean supply being processed into SBM (and oil) and nearly 97% of the resulting SBM used in livestock feeds (Kim et al., 2019). Meeting the increased demands for production of soybeans and other livestock feed ingredients leads to a reduction in arable land for other crops, emphasizing the importance in finding alternative, environmentally sustainable feed ingredients for livestock diets (Kim et al., 2019).

Insect larvae require less water, land, and resources to produce versus conventional plant-based feed ingredients (Wang and Shelomi, 2017). Black soldier fly larvae (BSFL; *Hermetia illucens*) can consume a variety of different substrates (e.g., pre- and post-consumer food products, distillers’ grains), while generating larvae biomass that is rich in protein and lipids, making BSFL meal (BSFLM) an attractive alternative protein source for inclusion in livestock diets (Spranghers et al., 2016; Altmann et al., 2019). Commonly dried after harvesting and ground into a meal, BSFLM contains, on average, 40% crude protein on a dry matter (DM) basis (Altmann et al., 2019), but the amino acid (AA) profile can be variable among BSFLM of different sources and processing techniques (De Marco et al., 2015; Mwaniki et al., 2018; Biasato et al., 2019).

Amino acid digestibility coefficients of BSFLM have been determined for fish (e.g., 90% apparent total tract digestibility of Lys for rainbow trout; De Marco et al., 2015) and poultry (e.g., 84% apparent ileal digestibility of Lys for broilers; Mwaniki and Kiarie, 2019), and the use of BSFLM in the diets of salmonids, trout, tilapia, and poultry (including chickens, ducks, turkey, and geese) has been approved by regulatory bodies (AAFCO, 2016). Conversely, the digestible AA and available energy in BSFLM have not been well characterized for pigs. Therefore, the objectives of this study were to determine the standardized ileal digestibility (SID) of crude protein (CP) and AA and net energy contents of full-fat (FF) and defatted (DF) BSFLM fed to growing pigs.

## MATERIALS AND METHODS

Animal care and use protocols were approved by the University of Guelph Animal Care and Use Committee (AUP #e3982). Pigs were cared for in accordance with the Canadian Council on Animal Care guidelines (CCAC, 2009).

### Black Soldier Fly Larvae Meal Samples and Experimental Diets

One full-fat (FF; Oreka Solutions, Markham, ON, Canada) and one defatted (DF) BSFLM (Enterra Feed Corporation; Langley, BC, Canada) sources were evaluated (Table 1). The FF and DF BSFLM were ground using a 0.6-mm screen size and the DF BSFLM was defatted via cold pressing to remove a portion of the lipids. Rearing and substrate materials used for both BSFLM sources were proprietary and not disclosed. Two corn-starch-based diets were then formulated to contain FF (50% inclusion level) and DF (36.5% inclusion level) BSFLM, respectively, as the sole source of AA and to achieve a target CP concentration of 20% (as-fed basis; Table 2). A nitrogen-free diet (N-F) was formulated according to Rho et al. (2017). The cornstarch:sucrose:oil ratio was kept constant among diets in order to calculate digestible energy (DE) by the difference method (Kiarie et al., 2016). Vitamins and minerals were added to all diets to meet or exceed estimated requirements for growing pigs (NRC, 2012), and titanium dioxide was included to determine nutrient and energy digestibilities (De Lange et al., 1998).

**Table 1.** Analyzed nutrient composition (as-fed basis) of full-fat (FF) and defatted (DF) black solider fly larvae meal

	Black soldier fly larvae meal	
Item	FF meal^1^	DF meal^2^
Dry matter, %	88.4	93.9
Crude protein, %	42.5	40.8
GE^3^, kcal/kg	5035	4256
Crude fat, %	32.5	12.8
Starch, %	0.8	14.0
Ash, %	7.42	6.80
Calcium, %	2.08	0.58
Phosphorus, %	0.47	0.76
Sodium, %	0.06	0.25
Potassium, %	0.61	1.28
Magnesium, %	0.25	0.33
NDF^3^, %	14.3	13.3
ADF^4^, %	7.7	9.1
NDF-N, %	9.8	5.8
Total dietary fiber, %	6.4	14.3
Indispensable AA^5^, %		
Arg	1.71	1.37
His	1.12	0.79
Ile	1.68	1.29
Leu	2.60	1.99
Lys	1.87	1.63
Met	0.75	0.77
Phe	1.65	1.15
Thr	1.25	1.05
Val	2.18	1.76
Dispensable AA, %		
Ala	2.20	2.13
Asp	3.31	2.68
Cys	0.21	0.39
Glu	3.50	3.99
Gly	2.18	1.61
Pro	2.16	2.04
Ser	1.44	1.31

^1^Full-fat BSFLM obtained from Oreka Solutions (Markham, ON, Canada).

^2^Defatted BSFLM obtained from Enterra Feed Corporation (Langley, BC, Canada).

^3^GE – gross energy.

^4^NDF = neutral detergent fiber.

^5^ADF = acid detergent fiber.

^6^AA = amino acid.

**Table 2.** Ingredient composition (%, as-fed basis) of the test diets

Ingredient, %	N-F diet^1^	FF diet^2^	DF diet^3^
Black solider fly larvae meal, full fat	—	50.00	—
Black solider fly larvae meal, defatted	—	—	36.50
Corn starch	84.80	39.21	51.52
Sucrose	6.17	2.85	3.75
Corn oil	2.06	0.95	1.25
Cellulose	2.06	2.06	2.06
Limestone	0.80	0.80	0.80
Monocalcium phosphate	2.30	2.30	2.30
NaCl	0.50	0.50	0.50
K_2_CO_3_	0.40	0.40	0.40
MgO	0.14	0.14	0.14
Vitamin and mineral premix^4^	0.60	0.60	0.60
TiO_2_	0.20	0.20	0.20
Calculated nutrient content, %^5^			
Crude protein	0.0*2*	21.11	19.36

^1^N-F diet = nitrogen-free basal diet.

^2^Full-fat BSFLM obtained from Oreka Solutions (Markham, ON, Canada).

^3^Defatted BSFLM obtained from Enterra Feed Corporation (Langley, BC, Canada).

^4^Provided, per kilogram of diet, 12,000 IU vitamin A as retinyl acetate, 1,200 IU vitamin D3 as cholecalciferol, 48 IU vitamin E as dl-α-tocopherol acetate, 3 mg vitamin K as menadione, 18 mg pantothenic acid, 6 mg riboflavin, 600 mg choline, 2.4 mg folic acid, 30 mg niacin, 18 mg thiamine, 1.8 mg pyridoxine, 0.03 mg vitamin B12, 0.24 mg biotin, 18 mg Cu from CuSO_4_∙5H_2_O, 120 mg Fe from FeSO_4_, 24 mg Mn from MnSO_4_, 126 mg Zn from ZnO, 0.36 mg Se from Na_2_SeO_3_, and 0.6 mg I from KI (DSM Nutritional Products Canada Inc., Ayr, ON, Canada).

^5^Calculated based on the NRC (2012) ingredient values with BSFLM ingredient values provided by Oreka Solutions (Markham, ON, Canada) and Enterra Feed Corporation (Langley, BC, Canada) for full fat and defatted BSFLM, respectively.

### Experiment 1

Eight barrows (Yorkshire × Landrace × Duroc; 25.1 kg BW ± 0.41 kg) were obtained from the University of Guelph Arkell Swine Research Station (Guelph, ON, Canada). All animals were housed individually in a temperature-controlled room (20 to 22 °C) for 7 d prior to surgery. Pigs were surgically fitted with a simple T-cannula at the distal ileum followed by a 1-wk postsurgical recovery period (De Lange et al., 1998). Each day, the surgical area was cleaned with warm water, dried thoroughly, and zinc oxide cream was applied. The experiment was conducted using a replicated 2 × 2 Latin square design (*n* = 8 over two experimental periods). At the beginning of each experimental period, pigs were weighed in order to determine feed allowance (2.8 × maintenance energy requirements; NRC, 2012). Pigs were fed either the FF diet or DF diet in meals at 0800 and 1700 h as a wet mash (water-to-feed ratio of approximately 2:1) and water was provided ad libitum from a low-pressure drinking nipple. Basal endogenous losses from Rho et al. (2017) were used to determine the SID of CP and AA (i.e. the N-F diet, animal genotype, and experimental design were identical); therefore, the N-F diet was not fed in Exp. 1. Each experimental period lasted for 7 d, with a 5-d adaptation period, followed by 2 d of continuous ileal digesta collection for 8 h after the morning meal. For ileal digesta collection, a bag was filled with 10 mL of 10% formic acid and was attached to the cannula using an elastic band and was replaced as needed. Digesta was kept in the refrigerator at 4 °C during collection, pooled per pig per period, and stored at −20 °C until analysis.

### Experiment 2

Exp. 2 was conducted at the same time as Exp. 1, in an identical room with the same environmental control and same feeding levels. Eight barrows (23.4 kg BW ± 0.54 kg) were randomly assigned to one of three experimental diets (Table 2) in a partially replicated Latin square design (*n* = 8 over three experimental periods). In each period, pigs were fed experimental diets once per day at 0800 h and were trained to consume the feed within 1 h. Remaining feed was removed after 1 h, dried, and weighed. Water was provided in troughs, which were filled twice per day. Each experimental period was 12 d where pigs were adjusted to the test diet for 7 d followed by 5 d in adjustable metabolism crates for total urine and fecal grab sample collection. Fresh fecal samples were collected twice daily, pooled per period per pig, and stored at −20 °C until analyses. Urine was filtered using glass wool and collected in buckets containing 10 mL of concentrated H_2_SO_4_ to minimize nitrogen losses. Ten percent of the daily urine weight was subsampled and stored at 4 °C until further analysis (Huber et al., 2013).

### Sample Preparation and Chemical Analyses

The ileal digesta and fecal samples were freeze-dried and finely ground prior to analysis. All samples, excluding urine, were analyzed for DM (AOAC, 2005; method 930.15). The BSFLM, experimental diets, fecal, digesta, and urine samples were analyzed for gross energy (GE) using a bomb calorimeter (IKA Calorimeter System C 5000; IKA Works Inc., Wilmington, NC) and N using a Foss Kjeltech 8200 Auto Distillation Unit (Fisher Scientific, Ottawa, ON) according to method 968.06 (AOAC, 2005). For urine GE, approximately 1 g of liquid urine was added to approximately 0.5 g of cellulose and freeze-dried. The urine–cellulose mixture was then analyzed using the bomb calorimeter and GE of urine was determined by subtracting the GE contribution from a cellulose blank. Experimental diets, fecal, and digesta samples were analyzed for titanium according to Myers et al. (2004) with minor adaptations (digestion for 24 h at 120 °C in 10 mL tubes and addition of H_2_O_2_ after precipitate settled in 100-mL volumetric flasks) and measured using a UV spectrophotometer. The experimental diets and ingredients were analyzed for calcium and phosphorus using inductively coupled plasma mass spectrophotometry (AOAC, 2005; method 985.01). The BSFLM, experimental diets, and fecal samples were analyzed for neutral detergent fiber (NDF) and acid detergent fiber (ADF) according to Van Soest et al. (1991) using an Ankom 200 Fiber Analyzer (Ankom Technology, Fairport, NY). The BSFLM sources were analyzed for NDF-N by performing N analysis, as described above, on the residual fraction after NDF analysis. Ethanol soluble carbohydrates and starch and total dietary fiber were analyzed in commercial laboratories (SGS Canada Inc, Guelph, ON, Canada and Intertek, Saskatoon, SK, Canada, respectively). The BSFLM, experimental diets, and ileal digesta were analyzed for AA using the performic acid oxidized hydrolysis procedure (AOAC, 2005; method 994.12), which allowed for the determination of Met and Cys but not Tyr or Trp. The AA were quantified via ion-exchange chromatography with post-column derivatization with ninhydrin according to Llames and Fontaine (1994).

### Calculations and Statistical Analysis

The apparent ileal digestibility (AID) and SID of CP and AA contents were calculated according to Stein et al. (2007). As indicated for Exp. 1, basal endogenous losses from Rho et al. (2017) were used to determine the SID of CP and AA. The apparent total tract digestibility (ATTD) of DE, NDF, and ADF in the BSFLM were calculated using the difference method (Adeola, 2001). The metabolizable energy (ME) contents of the FF and DF BSFLM were calculated by subtracting urinary energy excretion from the DE contents of the BSFLM. The predicted net energy (NE) of the test ingredients were calculated according to the equations established by Noblet et al. (1994):

$$\begin{array}{l} \begin{array}{l} NE\;=\;0.843\times DE\;\;-\;\;463,\\ NE\;=\;0.700\times DE\;+\;1.61\times \%\;crude\;fat\;+\;0.48\\ \;\times \%\;\;starch\;\;-\;\;0.91\times \%\;CP\;\;-\;\;0.87\times \%\;ADF,\\ NE\;=\;0.870\times ME\;\;-\;\;442,\;and\\ NE\;=\;0.726\times ME\;+\;1.33\times \%\;crude\;fat\;+\;0.39\\ \;\times \%\;starch\;\;-\;\;0.62\times \%\;CP\;\;-\;\;0.83\times \%\;ADF, \end{array} \end{array}$$

where NE, DE, and ME are in kilocalories per kg on a DM basis, and crude fat, starch, CP, and ADF are in percent DM. The average value of 4 prediction equations was used for the Noblet-predicted NE.

The predicted NE of the test ingredients were also calculated according to Blok et al. (2015) using the following equation:

$$\begin{array}{ll} NE\;= & \;((11.70\times digCP)\;+\;(35.74\times digCFat)\;\\ & +\;(14.14\times starch)\;+\;(9.74\times digNSP))/4.184, \end{array}$$

where NE is net energy in kilocalories per kg on a DM basis, digCP is the digestible crude protein content in grams per kilogram on a DM basis, digCFat is the digestible crude fat content in grams per kilogram on a DM basis, starch is in grams per kilogram on a DM basis, and digNSP is the digestible nonstarch polysaccharide (NSP) content in grams per kilogram on a DM basis. The digCFat content was estimated using:

$$\begin{array}{ll} digCFat\;(g/kg\;DM)\;= & \;0.9\times crude\;fat\;intake\;\\ & (g/kg\;DM)\;-\;5.0\;g/kg\;DM, \end{array}$$

and the digNSP was estimated using:

$$\begin{array}{l} digNSP\;(g/kg\;DM)\;=\;digOM\;-\;digCP\;-\;digCFat\;-\;starch, \end{array}$$

where digOM is the digestible organic matter in grams per kilogram on a DM basis, digCP is the digestible crude protein in grams per kilogram on a DM basis, digCFat is the digestible crude fat in grams per kilogram on a DM basis, and starch is in grams per kilogram on a DM basis. The digOM was estimated using:

$$\begin{array}{l} digOM\;(g/kg\;DM)=\;100\%-ash\%, \end{array}$$

where ash% was on a DM basis.

All data were analyzed using the GLIMMIX procedure of SAS (SAS Inst. Inc., Cary, NC). Individual pig was considered the experimental unit, diet and carryover between experimental periods were fixed effects, and period and diet fed within period were considered random effects. When appropriate, differences among individual means were assessed using the Tukey–Kramer post hoc test. A probability of *P* < 0.05 was considered significant, whereas 0.05 < *P* ≤ 0.10 was considered a tendency.

## RESULTS

All pigs remained healthy throughout the study, and accuracy of cannula placement in the terminal ileum was confirmed via necropsy at the conclusion of the study.

### Chemical Composition Experimental Diets and Ingredients

The analyzed chemical composition of FF and DF BSFLM samples are presented in Table 1. The calcium, NDF-N, crude fat, total dietary fiber, starch, sodium, and potassium were 259%, 69%, and 154% greater and 123%, 94%, 76%, and 52% less for FF than DF BSFLM, respectively. Generally, the analyzed AA concentrations for most AA in the FF BSFLM were approximately 18% greater than those of the DF BSFLM.

The ingredient composition and analyzed chemical composition of the experimental diets are presented in Tables 2 and 3, respectively. Test diets were formulated using the product specification sheets obtained from Oreka Solutions (Markham, ON, Canada) and Enterra Feed Corporation (Langley, BC, Canada) for the FF and DF BSFLM, respectively, to contain similar nutrient profiles (Table 3). The CP, crude fat, calcium, NDF, phosphorus, and potassium were 29%, 251%, 129%, and 20% greater and 17% and 60% less for the FF diet versus the DF BSFLM diet, respectively. The FF BSFLM diet provided approximately 30% more AA and 67% and 40% more Met and Asp, respectively, than the DF BSFLM diet.

**Table 3.** Analyzed nutrient composition (as-fed basis) of test diets

	Treatment		
Item	FF diet^1^	DF diet^2^	N-F diet^3^
Dry matter, %	88.8	91.7	90.8
Crude protein, %	21.5	16.7	0.85
Crude fat, %	17.2	4.9	—
Calcium, %	1.76	0.77	0.72
Phosphorus, %	0.57	0.69	0.25
Sodium, %	0.26	0.25	0.16
Potassium, %	0.32	0.81	0.14
Magnesium, %	0.16	0.16	0.06
NDF^4^, %	10.3	8.6	1.9
ADF^5^, %	4.8	4.7	0.6
Indispensable AA, %			
Arg	0.88	0.64	0.02
His	0.54	0.40	0.01
Ile	0.78	0.60	0.02
Leu	1.32	0.95	0.06
Lys	0.99	0.73	0.02
Met	0.40	0.24	0.01
Phe	0.78	0.58	0.03
Thr	0.69	0.51	0.02
Val	1.02	0.81	0.03
Dispensable AA^6^, %			
Ala	1.12	0.99	0.04
Asp	1.74	1.24	0.05
Cys	0.17	0.27	0.02
Glu	1.87	1.84	0.09
Gly	1.03	0.77	0.03
Pro	1.06	0.95	0.05
Ser	0.76	0.57	0.03

^1^Full fat BSFLM obtained from Oreka Solutions (Markham, ON, Canada).

^2^Defatted BSFLM obtained from Enterra Feed Corporation (Langley, BC, Canada).

^3^Indispensable and dispensable AA % obtained from Rho et al. (2017) as N-F diets were formulated identically.

^4^NDF = neutral detergent fiber.

^5^ADF = acid detergent fiber.

^6^AA = amino acid.

### Apparent and Standardized Ileal Digestibility of CP and AA

The AID of CP and AA for FF BSFLM were generally not different versus DF BSFLM (Table 4), except the FF BSFLM had greater AID for Met and lower AID for Val, Ala, and Cys than DF BSFLM (*P* < 0.05). The SID of CP and AA for FF BSFLM were generally not different versus DF BSFLM (Table 5), except the FF BSFLM had lower SID for Arg (*P* < 0.05) and tended to have lower SID for Val (*P* = 0.090), Ala (*P* = 0.053), and Pro (*P* = 0.065) and greater SID for Met (*P* = 0.074) compared with the DF BSFLM.

**Table 4.** Apparent ileal digestibility (%) of crude protein (CP) and amino acid (AA) in full fat (FF meal) and defatted (DF meal) black soldier fly larvae meal samples (*n* = 8) fed to growing pigs (Exp. 1)

	Black soldier fly larvae meal			
Item	FF meal^1^	DF meal^2^	SEM^3^	*P*-value
CP	71.2	71.0	1.45	0.932
Indispensable AA				
Arg	86.3	86.2	1.29	0.956
His	75.0	77.5	2.65	0.454
Ile	80.7	83.2	1.58	0.303
Leu	80.6	83.4	1.59	0.251
Lys	80.9	82.2	1.63	0.561
Met	87.4	74.1	3.54	0.031
Phe	85.2	84.2	1.58	0.640
Thr	75.4	73.5	2.13	0.557
Val	75.6	81.9	1.88	0.049
Dispensable AA				
Ala	78.4	83.5	1.29	0.031
Asp	81.7	79.1	1.87	0.365
Cys	66.8	75.3	2.06	0.035
Glu	80.9	78.3	1.92	0.388
Gly	61.6	65.7	2.86	0.336
Pro	73.2	77.7	1.62	0.093
Ser	75.1	76.4	1.68	0.624

^1^Full fat BSFLM obtained from Oreka Solutions (Markham, ON, Canada).

^2^Defatted BSFLM obtained from Enterra Feed Corporation (Langley, BC, Canada).

^3^Maximum value of SEM.

**Table 5.** Standardized ileal digestibility (%) of crude protein (CP) and amino acid (AA) in full fat (FF meal) and defatted (DF meal) black soldier fly larvae meal samples (*n* = 8) compared with SBM fed to growing pigs (Exp. 1)

	Black soldier fly larvae meal				
Item	FF meal^1^	DF meal^2^	SEM^3^	*P*-value	SBM^4^
CP	80.2	81.0	1.11	0.579	87
Indispensable AA					
Arg	92.7	95.9	1.05	0.034	94
His	80.7	84.3	2.56	0.185	90
Ile	87.2	89.6	1.34	0.253	89
Leu	87.2	90.7	1.43	0.130	88
Lys	86.8	89.1	1.41	0.250	89
Met	90.2	79.3	3.61	0.074	90
Phe	95.4	97.6	1.42	0.297	88
Thr	87.2	86.6	1.92	0.829	85
Val	83.2	88.4	1.80	0.090	87
Dispensable AA					
Ala	85.7	89.9	1.26	0.053	85
Asp	87.9	86.6	1.71	0.625	87
Cys	86.0	83.1	2.14	0.418	84
Glu	87.5	84.2	1.81	0.247	89
Gly	81.4	85.1	2.47	0.258	84
Pro	100.1	104.6	1.57	0.065	113
Ser	85.5	88.1	1.55	0.289	89

^1^Full fat BSFLM obtained from Oreka Solutions (Markham, ON, Canada).

^2^Defatted BSFLM obtained from Enterra Feed Corporation (Langley, BC, Canada).

^3^Maximum value of SEM.

^4^SBM SID% of CP and AA from NRC (2012) for reference only.

### Nitrogen Balance and Apparent Total Tract Digestibility of GE, NDF, and ADF

There were no differences in N intake, output in feces, output in urine, retained (% or g/d), or ATTD for pigs fed the FF versus DF BSFLM diets (Table 6). The ATTD of NDF and ADF were greater for FF than DF BSFLM (*P* = 0.052 and *P* < 0.05, respectively; Table 7), but the ATTD of GE was not different between the FF and DF BSFLM.

**Table 6.** Nitrogen balance of full fat (FF diet) and defatted (DF diet) black soldier fly larvae meal diets (*n* = 8) fed to growing pigs (Exp. 2)

	Treatment			
Item	FF diet^1^	DF diet^2^	SEM^3^	*P*-value
Nitrogen balance				
N intake, g/d	31.8	28.0	2.39	0.119
N output in feces, g/d	6.5	5.8	0.50	0.436
N excretion in urine, g/d	7.6	8.6	1.57	0.466
N retained, %	56.1	48.2	3.73	0.166
N retained, g/d^4^	18.2	13.1	1.56	0.280
N digestibility, %^5^	80.1	78.7	1.40	0.602

^1^Full fat BSFLM obtained from Oreka Solutions (Markham, ON, Canada).

^2^Defatted BSFLM obtained from Enterra Feed Corporation (Langley, BC, Canada).

^3^Maximum value of SEM.

^4^N retained (g/d) = N intake (g/d) − N output in feces (g/d) − N output in urine (g/d).

^5^N digestibility (%) = 100 − (100 × ((Diet Ti (mg/kg) × N output fecal (%))/(Sample Ti (mg/kg) × Diet N (%)))).

**Table 7.** Apparent total tract digestibility (ATTD, %) of chemical components in full fat (FF meal) and defatted (DF meal) black soldier fly larvae meal samples (*n* = 8) fed to growing pigs (Exp. 2)

	BSFLM^1^			
Item^2^	FF meal^1^	DF meal^2^	SEM^3^	*P*-value
ATTD, %				
GE	76.0	74.6	1.60	0.528
NDF	86.1	76.1	2.71	0.052
ADF	91.0	69.5	5.03	0.023

^1^BSFLM = Black soldier fly larvae meal.

^2^GE = gross energy; NDF = neutral detergent fiber; ADF = acid detergent fiber.

^3^Full-fat BSFLM obtained from Oreka Solutions (Markham, ON, Canada).

^4^Defatted BSFLM obtained from Enterra Feed Corporation (Langley, BC, Canada).

^5^Maximum value of SEM.

### Available Energy

The FF diet had greater DE and ME than the DF and N-F diets (*P* < 0.001; Table 8). The FF BSFLM had greater GE, DE, ME, and predicted NE contents than DF BSFLM (regardless of prediction method; *P* < 0.05).

**Table 8.** Energy values of test diets and ingredients (*n* = 8; Exp. 2)

	Treatment				
Item^1^	FF diet^2^	DF diet^3^	N-F diet^4^	SEM^5^	*P*-value
Energy value, kcal/kg DM					
GE	5,163	4,473	4,106	—	—
DE	4,431^a^	3,918^b^	3,870^b^	31	<0.001
ME	4,187^a^	3,608^b^	3,718^b^	40	<0.001
Energy value of Insect meal products, kcal/kg DM					
GE	5,696	4,533	—	336	0.026
DE	4,927	3,941	—	75	<0.001
ME	4,569	3,396	—	102	<0.001
Predicted NE Noblet^5^	3,477	2,640	—	30	<0.001
Predicted NE Blok^6^	3,479	2,287	—	28	<0.001

^a,b^Means within a row with different superscripts differ (*P* < 0.05; *n* = 8).

^1^DM = dry matter; GE = gross energy; DE = digestible energy; ME = metabolizable energy; NE = net energy.

^2^Full-fat black soldier fly larvae meal (BSFLM) obtained from Oreka Solutions (Markham, ON, Canada).

^3^Defatted BSFLM obtained from Enterra Feed Corporation (Langley, BC, Canada).

^4^N-F diet = nitrogen-free basal diet.

^5^Maximum value of SEM.

^6^The average of four predicted NE from Noblet et al. (1994), where 1) NE = 0.843 × DE − 463 (NE of FF and DF BSFLM were 3,695 and 2,855 kcal/kg DM, respectively); 2) NE = 0.700 × DE + 1.61 × % crude fat + 0.48 × % starch − 0.91 × % CP − 0.87 × % ADF (NE of FF and DF BSFLM were 3,459 and 2,739 kcal/kg DM, respectively); 3) NE = 0.870 × ME − 442 (NE of FF and DF BSFLM were 3,548 and 2,498 kcal/kg DM, respectively); and 4) NE = 0.726 × ME + 1.33 × % crude fat + 0.39 × % starch − 0.62 × % CP − 0.83 × % ADF (NE of FF and DF BSFLM were 3,233 and 2,443 kcal/kg DM, respectively).

^6^Using predicted NE from Blok et al. (2015), where NE kcal/kg = ((11.70 × digCP) + (35.74 × digCFat) + (14.14 × Starch) + (9.74 × digNSP))/4.184.

## DISCUSSION

The current study demonstrated that both FF and DF BSFLM are reasonable sources of digestible Lys for growing pigs, with SID of Lys (i.e., 88%; average for FF and DF BSFLM) generally at least as high as for SBM (i.e., SID Lys of 89%; NRC, 2012), as well as those for animal-based protein sources such as blood meal (i.e., SID Lys of 84%; Kerr et al., 2019) and fishmeal (i.e., SID Lys of 86%; NRC, 2012). However, the Lys and CP concentrations for both FF and DF BSFLM were approximately 40% and 13% less than those of SBM, respectively. This suggests that these sources of BSFLM provide less digestible Lys and CP overall. In addition, for some AA (namely Arg, Val, Ala, and Pro) the SID were, or tended to be, less for the FF versus the DF BSFLM, which could be attributed to the relatively greater NDF-N concentration in FF BSFLM. The fibrous fraction of BSFLM is found in chitin, which is a major component of the larvae exoskeleton (Wang and Shelomi, 2017). The β1–4 bonds between the N-acetylglucosamine subunits that compose chitin are not digested by endogenous enzymes (Huang et al., 2001). Therefore, the N within the chitin polymer and any protein encapsulated by chitin would not be digested and absorbed within the small intestine of the pig. Furthermore, some differences in SID of AA between the FF and DF BSFLM may be attributed to different processing methods applied to BSFLM (Gravel and Doyen, 2020). The use of cold pressing to defat BSFLM has minimal effects on AID in broiler chickens (Schiavone et al., 2017). However, BSFL are also typically dried prior to grinding (Gravel and Doyen, 2020). Huang et al. (2018) found that the in vitro digestibility of AA were greater when BSFLM was dried using conventional methods (at 60 °C to constant weight) versus drying with microwave irradiation (an increasingly popular drying method used in food processing). Though the BSFLM suppliers for the current study did not disclose the drying processes used, this may be a potential source of variation for the SID of AA in FF versus DF BSFLM. Despite some small differences in SID of AA, whole body N utilization was not different between pigs fed FF and DF BSFLM. This indicates that the absorbed AA from the two BSFLM sources were used equally to support protein retention in growing pigs, when included in the diets at 50% and 36.5%, respectively, as the sole sources of dietary AA.

In the current study, the calcium concentration of the FF BSFLM was approximately 4× greater than that of the DF BSFLM, whereas the phosphorus concentration of the FF BSFLM was approximately 40% less than that of the DF BSFLM. Spranghers et al. (2016) determined that calcium concentration in BSFLM is highly variable and dependent on larvae rearing substrate, which could explain the differences in calcium concentration for the FF versus DF BSFLM sources. Conversely, the same research group also demonstrated that phosphorus concentration of BSFLM was less dependent on rearing substrates (Spranghers et al., 2016). Though determining ATTD and standardized total tract digestibilities of minerals was beyond the scope of the current study, it is well established that the digestibility of calcium and phosphorus are generally greater for animal sources versus plant sources (Suttle, 2010). If the calcium and phosphorus in insect larvae are also highly digestible and BSFL can consistently be enriched with calcium (and phosphorus) by targeting appropriate rearing substrates, BSFLM may also be used as a source of these macro-minerals in swine diets. It is noted, however, that the ratio of calcium-to-phosphorus can be variable between BSFLM sources (Table 1), and care should be taken when formulating swine diets to ensure appropriate calcium-to-phosphorus ratios in the complete feed to avoid mineral antagonisms (Stein et al., 2011). This will require strict quality control of BSFLM by suppliers.

In the current study, the NE of the FF and BSFLM samples were predicted using two different methods. The predicted NE for FF BSFLM using the equations established by Noblet et al. (1994) and Blok et al. (2015) were in close alignment. The predicted NE values for the DF BSFLM determined using the equation from Blok et al. (2015) however, were 13% less than the predicted NE value using the equations from Noblet et al. (1994). The key differences between the two NE prediction methods was that Blok et al. (2015) accounted for the digestible components of the ingredient, including NSP, whereas Noblet et al. (1994) only accounted for ADF, DE or ME, and analyzed crude nutrient contents. Furthermore, the DF BSFLM contained 123% greater total dietary fiber than the FF BSFLM, which could reduce NE (vs. starch and fat) since fiber increases the heat increment of feeding to a greater extent than either starch or fat (Noblet and Henry, 1993). It is likely that the combination of accounting for digestible components and the high fiber content in DF BSFLM contribute to the differences in predicted NE between the Noblet et al. (1994) and Blok et al. (2015) methods for DF BSFLM.

From an energetics standpoint, FF BSFLM was a better source of net energy for growing pigs compared to DF BSFLM, which is likely due to the greater concentration of (crude) fat in the FF BSFLM (32.5% vs. 12.8% in FF and DF BSFLM, respectively). Since fat is highly digestible and has a low heat increment of feeding (Stahly, 1984), this result is expected. The increased fat content in FF BSFLM may also have an effect on the ATTD of NDF and ADF. Indeed, the FF BSFLM had greater ATTD of NDF and ADF compared to the DF BSFLM (86.1% and 91.0% vs. 76.1% and 69.5% for ATTD of NDF and ADF for FF BSFLM and DF BSFLM, respectively). Dégen et al. (2009) also demonstrated that greater dietary fat content improved the ATTD of fiber. In addition, the predicted NE of the FF BSFLM was in the range of NE supplied by full-fat sunflower meal (i.e., 3,678 kcal/kg DM; NRC, 2012), whereas the NE of the DF BSFLM was in the range of NE supplied by animal protein sources such as blood plasma, fish meal, and whey powder (e.g., 2,725, 2,509, and 2,783 kcal/kg DM for blood plasma, fish meal, and whey powder, respectively; NRC, 2012). Therefore, in addition to providing digestible AA, BSFLM, and especially FF BSFLM, can also serve as a source of NE in swine diets.

The ability of BSFL to use organic waste products as a substrate makes them an attractive substitute for some other, less sustainable, protein sources in swine diets. Moreover, BSFL can create 1 kg of larvae biomass per 2 kg of rearing substrate (Makkar et al., 2014), in order to generate a value-added product from organic waste, while simultaneously reducing organic matter sent to landfill. Despite these anticipated environmental benefits of BSFLM, it is also necessary to consider ingredient cost and logistics (e.g., production capacity in North America). Currently, the cost of BSFLM precludes its use in livestock diets as a protein source (Biasato et al., 2019). For example, on a per kg basis, FF BSFLM would have to be approximately half the price of SBM in order to supply SID Lys at the same cost, based on the relative difference in SID Lys supply between FF BSFLM and SBM. Therefore, it may be more advantageous to focus on BSFLM as a feed additive versus as an alternative protein source in swine diets.

Practically, the use of BSFLM may be most suitable for use in nursery diets for newly weaned pigs. The BSFL contains high amounts of lauric acid [a medium chain fatty acid (MCFA)] constituting up to 70% of the total saturated fatty acid content (depending on rearing substrate; Spranghers et al., 2016; Surendra et al., 2016; Barragan-Fonseca et al., 2017). Lauric acid, and its metabolites, have antimicrobial and anti-inflammatory properties in the small intestine (Devi and Kim, 2014; Spranghers et al., 2016). In addition, BSFL contains chitin, which is a prebiotic and known to increase the richness and diversity of beneficial gut microflora in laying hens compared with standard diets without BSFLM (Borrelli et al., 2017). Together, the mono- and poly-unsaturated fatty acids, MCFA, and chitin could promote increased nutrient absorption and reduce colonization of pathogenic bacteria in the gut of the newly weaned pig (Fioramonti et al., 2003; Heo et al., 2013), making BSFLM and its fractions attractive alternatives for in-feed antimicrobials during the post-weaning period. Future research should explore and optimize the functional benefits of BSFLM and its fractions in nursery diets.

In summary, the findings of the present study suggest that both FF and DF BSFLM could be suitable protein alternatives for inclusion in swine diets. Both FF and DF BSFLM had high SID for most AA, despite providing less digestible protein overall compared to SBM. The FF BSFLM was a better source of available energy for growing pigs, whereas both the FF and DF BSFLM had predicted NE contents comparable to various full-fat oilseed meals and animal proteins, respectively, fed to pigs. Furthermore, this study provides data that will be useful for the accurate formulation of swine diets including BSFLM. At this time however, BSFLM is cost-prohibitive for inclusion in livestock diets due to lack of infrastructure to support cost-effective production. Therefore, it may be more appropriate to explore other functional benefits of BSFLM and its fractions versus using them as protein alternatives in swine diets until the infrastructure exists to produce BSFL on a larger scale.

## References

[CIT0001] AAFCO. 2016 Association of American feed control officials. In: Proceedings of the AAFCO Annual Meeting Agenda and Committee Reports, Pittsburgh, PA, 31 July–3 August 2016 AAFCO: Pittsburgh, PA p. 112.

[CIT0002] Adeola O 2001 Digestion and balance techniques in pigs. In: Lewis A. J. and Southern L. L., editors, Swine nutrition no. 2. CRC Press LLC, Boca Raton, FL p. 903–916.

[CIT0003] Altmann B. A., Neumann C., Rothstein S., Liebert F., and Mörlein D.. 2019 Do dietary soy alternatives lead to pork quality improvements or drawbacks? A look into micro-alga and insect protein in swine diets. Meat Sci. 153:26–34. doi:10.1016/j.meatsci.2019.03.001 30861487

[CIT0004] AOAC 2005 Official methods of analysis of AOAC International. AOAC Int., Gaithersburg, MD.

[CIT0005] Barragan-Fonseca K., Dicke M., and Loon J. V.. 2017 Nutritional value of the black soldier fly (*Hermetia illucens* L.) and its suitability as animal feed – A review. J. Insects as Food Feed. 2:105–120. doi:10.3920/jiff2016.0055

[CIT0006] Biasato I., Renna M., Gai F., Dabbou S., Meneguz M., Perona G., Martinez S., Lajusticia A. C. B., Bergagna S., Sardi L., et al. 2019 Partially defatted black soldier fly larva meal inclusion in piglet diets: effects on the growth performance, nutrient digestibility, blood profile, gut morphology and histological features. J. Anim. Sci. Biotechnol. 10:12. doi:10.1186/s40104-019-0325-x 30820321 PMC6379995

[CIT0007] Blok M. C., Brandsma G., Bosch G., Gerrits W. J. J., Jansman A. J. M., Fledderus J., and Everts H.. 2015 A new Dutch net energy formula for feed and feedstuffs for growing and fattening pigs. CVB-Documentation Report No. 56, Wageningen UR Livestock Research, Wageningen, The Netherlands.

[CIT0008] Borrelli L., Coretti L., Dipineto L., Bovera F., Menna F., Chiariotti L., Nizza A., Lembo F., and Fioretti A.. 2017 Insect-based diet, a promising nutritional source, modulates gut microbiota composition and SCFAs production in laying hens. Sci. Rep. 7:16269. doi:10.1038/s41598-017-16560-6 29176587 PMC5701250

[CIT0010] Canadian Council on Animal Care (CCAC). 2009 Guidelines on: The care and use of farm animals in research, teaching and testing. CCAC, Ottawa, ON, Canada.

[CIT0011] Dégen L., Halas V., Tossenberger J., Szabó C., and Babinszky L.. 2009 The impact of dietary fiber and fat levels on total tract digestibility of energy and nutrients in growing pigs and its consequence for diet formulation. Acta Agric. Scand. A Anim. Sci. 59:150–160. doi:10.1080/09064700903254281

[CIT0012] De Lange C. F. M., Gabert V. M., Gillis D., and Patience J. F.. 1998 Digestible energy contents and apparent ileal amino acid digestibilities in regular or partial mechanically dehulled canola meal samples fed to growing pigs. Can. J. Anim. Sci. 78:641–648. doi:10.4141/A97-114

[CIT0013] De Marco M., Martínez S., Hernandez F., Madrid J., Gai F., Rotolo L., Belforti M., Bergero D., Katz H., Dabbou S., et al. 2015 Nutritional value of two insect larval meals (*Tenebrio molitor* and *Hermetia illucens*) for broiler chickens: apparent nutrient digestibility, apparent ileal amino acid digestibility and apparent metabolizable energy. Anim. Feed Sci. Technol. 209:211–218. doi:10.1016/j.anifeedsci.2015.08.006

[CIT0014] Devi S. M. and Kim I.. 2014 Effect of medium chain fatty acids (MCFA) and probiotic (Enterococcus faecium) supplementation on the growth performance, digestibility and blood profiles in weanling pigs. Vet. Med.-Czech. 11:527–535. doi:10.17221/7817-vetmed

[CIT0015] Fioramonti J., Theodorou V., and Bueno L.. 2003 Probiotics: what are they? What are their effects on gut physiology? Best Pract. Res. Clin. Gastroenterol. 17:711–724. doi:10.1016/s1521-6918(03)00075-1 14507583

[CIT0016] Gravel A., and Doyen A.. 2020 The use of edible insect proteins in food: challenges and issues related to their functional properties. Innov. Food Sci. Emerg. Technol. 59:102272. doi:10.1016/j.ifset.102272

[CIT0017] Heo J. M., Opapeju F. O., Pluske J. R., Kim J. C., Hampson D. J., and Nyachoti C. M.. 2013 Gastrointestinal health and function in weaned pigs: A review of feeding strategies to control post-weaning diarrhoea without using in-feed antimicrobial compounds. J. Anim. Physiol. Anim. Nutr. (Berl). 97:207–237. doi:10.1111/j.1439-0396.2012.01284.x 22416941

[CIT0018] Huang C., Feng W., Xiong J., Wang T., Wang W., Wang C., and Yang F.. 2018 Impact of drying method on the nutritional value of the edible insect protein from black soldier fly (*Hermetia illucens* L.) larvae: Amino acid composition, nutritional value evaluation, in vitro digestibility, and thermal properties. Eur. Food Res. Technol. 1:11–21. doi:10.1007/s00217-018-3136-y

[CIT0019] Huang S. X., Sauer W. C., and Marty B.. 2001 Ileal digestibilities of neutral detergent fiber, crude protein, and amino acids associated with neutral detergent fiber in wheat shorts for growing pigs. J. Anim. Sci. 79:2388–2396. doi:10.2527/2001.7992388x 11583425

[CIT0020] Huber L., Squires E. J., and de Lange C. F.. 2013 Dynamics of nitrogen retention in entire male pigs immunized against gonadotropin-releasing hormone. J. Anim. Sci. 91:4817–4825. doi:10.2527/jas.2013-6290 23989879

[CIT0021] Kerr B. J., Urriola P. E., Jha R., Thomson J. E., Curry S. M., and Shurson G. C.. 2019 Amino acid composition and digestible amino acid content in animal protein by-product meals fed to growing pigs. J. Anim. Sci. 11:4540–4547. doi:10.1093/jas/skz294 PMC6827409 31587052

[CIT0022] Kiarie E., Walsh M. C., He L., Velayudhan D. E., Yin Y. L., and Nyachoti C. M.. 2016 Phytase improved digestible protein, phosphorous, and energy contents in camelina expellers fed to growing pigs. J. Anim. Sci. 94:215–218. doi:10.2527/jas.2015-9735

[CIT0023] Kim S. W., Less J. F., Wang L., Yan T., Kiron V., Kaushik S. J., and Lei X. G.. 2019 Meeting global feed protein demand: challenge, opportunity, and strategy. Annu. Rev. Anim. Biosci. 1:221–243. doi:10.1146/annurev-animal-030117-014838 30418803

[CIT0025] Llames C. R., and Fontaine J.. 1994 Determination of amino acids in feeds: Collaborative study. J AOAC Int. 77:1362–1402.

[CIT0026] Makkar H. P., Tran G., Heuzé V., and Ankers P.. 2014 State-of-the-art on use of insects as animal feed. Anim. Feed Sci. Technol. 197:1–33. doi:10.1016/j.anifeedsci.2014.07.008

[CIT0027] Mwaniki Z., and Kiarie E.. 2019 Standardized ileal digestible amino acids and apparent metabolizable energy content in defatted black soldier fly larvae meal fed to broiler chickens. Can. J. Anim. Sci. 2:211–217. doi:10.1139/cjas-2018-0111

[CIT0028] Mwaniki Z., Neijat M., and Kiarie E.. 2018 Egg production and quality responses of adding up to 7.5% defatted black soldier fly larvae meal in a corn–soybean meal diet fed to Shaver White Leghorns from wk 19 to 27 of age. Poult. Sci. 8:2829–2835. doi:10.3382/ps/pey118 PMC6044407 29669038

[CIT0029] Myers W. D., Ludden P. A., Nayigihugu V., and Hess B. W.. 2004 Technical note: A procedure for the preparation and quantitative analysis of samples for titanium dioxide. J. Anim. Sci. 82:179–183. doi:10.2527/2004.821179x 14753360

[CIT0030] Noblet J., Fortune H., Shi X. S., and Dubois S.. 1994 Prediction of net energy value of feeds for growing pigs. J. Anim. Sci. 72:344–354. doi:10.2527/1994.722344x 8157519

[CIT0031] Noblet J., and Henry Y.. 1993 Energy evaluation systems for pig diets: A review. Livest. Prod. Sci. 36:121–141. doi:10.1016/0301-6226(93)90147-a

[CIT0032] NRC. 2012 Nutrient requirements of swine, 11th edn. National Academy Press, Washington, DC.

[CIT0033] Rho Y., Zhu C., Kiarie E., and de Lange C. F. M.. 2017 Standardized ileal digestible amino acids and digestible energy contents in high-protein distillers dried grains with solubles fed to growing pigs. J. Anim. Sci. 8:3591–3597. doi:10.2527/jas.2017.1553 28805905

[CIT0034] Schiavone A., Marco M. D., Martínez S., Dabbou S., Renna M., Madrid J., Hernandez F., Rotolo L., Costa P., Gai F., et al. 2017 Nutritional value of a partially defatted and a highly defatted black soldier fly larvae (*Hermetia illucens* L.) meal for broiler chickens: Apparent nutrient digestibility, apparent metabolizable energy and apparent ileal amino acid digestibility. J. Anim. Sci. Biotechnol. 8:51. doi:10.1186/s40104-017-0181-5 28603614 PMC5465574

[CIT0035] Spranghers T., Ottoboni M., Klootwijk C., Ovyn A., Deboosere S., Meulenaer B. D., Michiels J., Eeckhout M., Clercq P. D., and Smet S. D.. 2016 Nutritional composition of black soldier fly (*Hermetia illucens*) prepupae reared on different organic waste substrates. J. Sci. Food Agric. 8:2594–2600. doi:10.1002/jsfa.8081 27734508

[CIT0036] Stahly T. S 1984 Use of fats in diets for growing pigs. In: J. Wiseman, editor. Fats in animal nutrition. London (UK): Butterworths; p. 313–331.

[CIT0037] Stein H. H., Adeola O., Cromwell G. L., Kim S. W., Mahan D. C., and Miller P. S.. 2011 Concentration of dietary calcium supplied by calcium carbonate does not affect the apparent total tract digestibility of calcium, but decreases digestibility of phosphorus by growing pigs. J. Anim. Sci. 7:2139–2144. doi:10.2527/jas.2010-3522 21335534

[CIT0038] Stein H. H., Seve B., Fuller M. F., Moughan P. J., and de Lange C. F. M.. 2007 Invited review: Amino acid bioavailability and digestibility in pig feed ingredients: terminology and application. J. Anim. Sci. 85:172–180. doi:10.2527/jas.2005-742 17179553

[CIT0039] Surendra K., Olivier R., Tomberlin J. K., Jha R., and Khanal S. K.. 2016 Bioconversion of organic wastes into biodiesel and animal feed via insect farming. Renew. Energy 98:197–202. doi:10.1016/j.renene.2016.03.022

[CIT0040] Suttle N. F 2010 The mineral nutrition of livestock. 4th ed. CABI Publishing, Wallingford, UK.

[CIT0041] Van Soest P. J., Robertson J. B., and Lewis B. A.. 1991 Methods for dietary fiber, neutral detergent fiber, and nonstarch polysaccharides in relation to animal nutrition. J. Dairy Sci. 74:3583–3597. doi:10.3168/jds.S0022-0302(91)78551-2 1660498

[CIT0042] Wang Y.-S., and Shelomi M.. 2017 Review of black soldier fly (*Hermetia illucens*) as animal feed and human food. Foods 10:91. doi:10.3390/foods6100091 PMC5664030 29057841

